# Interleukin‐33 as an early predictor of cetuximab treatment efficacy in patients with colorectal cancer

**DOI:** 10.1002/cam4.4331

**Published:** 2021-10-19

**Authors:** Xujun Zhang, Kefan Bi, Xiaoxuan Tu, Qiong Zhang, Qingyi Cao, Yan Liang, Ping Zeng, Lin Wang, Tianxing Liu, Weijia Fang, Hongyan Diao

**Affiliations:** ^1^ State Key Laboratory for Diagnosis and Treatment of Infectious Diseases National Clinical Research Center for Infectious Diseases Collaborative Innovation Center for Diagnosis and Treatment of Infectious Diseases The First Affiliated Hospital Zhejiang University School of Medicine Hangzhou China; ^2^ Department of Medical Oncology First Affiliated Hospital Zhejiang University School of Medicine Hangzhou China; ^3^ Department of Biological Sciences University of Toronto Toronto Ontario Canada

**Keywords:** cetuximab, colorectal cancer, IL‐33, NK cell

## Abstract

**Background:**

Cetuximab is used for colorectal cancer (CRC) treatment. However, the early biomarker of treatment efficacy of cetuximab has not been identified.

**Methods:**

After 1 year of cetuximab treatment, patients were divided into an effective group and an ineffective group. The interleukin‐33 (IL‐33) level and the distribution of lymphatic cells in patients were investigated by analyzing the peripheral blood mononuclear cells via flow cytometry analysis and ELISA. The correlation between IL‐33 immunomodulatory effect and cetuximab treatment efficacy was determined through experiments in vivo and in vitro.

**Results:**

The IL‐33 level in the peripheral blood was increased at 4 weeks after cetuximab administration of effective group, meanwhile, the osteopontin (OPN) was reduced. Whereas neither IL‐33 level nor OPN level of ineffective patients changed. In the effective group, the number of natural killer (NK) and CD8^+^ T cells were increased. Moreover, CD137 and CD107a expression on NK cells were higher in the effective group compared to the ineffective group. In vitro cetuximab treatment also increased the number of NK and CD8^+^ T cells as well as CD137 and CD107a expression upon IL‐33 stimulation. Moreover, the secretion of OPN was inhibited by IL‐33 administration in cetuximab‐treated PBMCs from the effective group patients. IL‐33 upregulated the cytotoxicity of NK cells and inhibited tumor cells growth in the effective cetuximab treatment mice.

**Conclusion:**

Effective cetuximab treatment induced a change of IL‐33 and OPN at the early stage and triggered the NK cells antitumor activity. Consequently, significantly increased IL‐33 level and decreased OPN level in the peripheral blood at the early treatment are proposed as potential predictors of cetuximab treatment efficacy.

## Graphical abstract

Cetuximab is widely used for metastatic colorectal cancer (CRC) treatment. However, biomarker for the efficacy of cetuximab has not been identified yet. Here, according to the efficacy of the cetuximab treatment after 12 months, CRC patients were divided into the effective group and the ineffective group. IL‐33 levels of ineffective patients were significantly increased in the peripheral blood at 4 weeks after cetuximab administration. We further revealed that IL‐33 promoted the cytotoxicity of NK cells through the upregulation CD137 and CD107a expression. Effective cetuximab treatment induced an increase of IL‐33 at the early stage and promoted the cytotoxic activity of NK cells to counter against tumor cells. Consequently, significantly increased IL‐33 level in the peripheral blood at the early treatment is proposed as a potential predictor of cetuximab treatment efficacy.

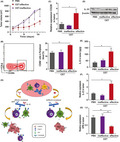

## 1 INTRODUCTION

Colorectal cancer (CRC)^1^, ^2^ is prevalent worldwide and has a high mortality rate. Surgical treatment is the main method to treat CRC; however, after initial surgery, approximately 40% patients will relapse within the few years.^3^ Cetuximab belongs to the chimeric anti‐epidermal growth factor receptor (EGFR) immunoglobulin (Ig) G1 monoclonal antibody. It has already been developed for inhibiting the proliferation, and metastasis of tumor cells.^4^, ^5^, ^6^ More recently, cetuximab was approved for the first‐line treatment in advanced CRC according to the US Food and Drug Administration.^7^, ^8^ Cetuximab is often prescribed for a long time to achieve a desired efficacy. However, in some patients, their condition does not improve after long‐term cetuximab treatment, which puts the patients under pressure of missing the ideal time for treatment, as well as increasing their financial burden. As a result, it has become vital to develop a biomarker to evaluate the efficacy of cetuximab at an early stage.

NK cells and CD8^+^ T cells play a significant role in the treatment of cetuximab.^9^ Interleukin‐33 (IL‐33) belongs to the IL‐1 family and is detectable in a variety of cells, including fibroblasts, epithelial cells, and endothelial cells. IL‐33 is identical to a nuclear factor preferentially expressed in high endothelial venules (HEV).^10^, ^11^ After tissue damage and necrosis, IL‐33 can function as an “alarm.”^12^ However, it might also be released from living cells and the immune response in a variety of autoimmune disorders. A positive relationship was also identified between IL‐33 and cetuximab in tumors. Many studies have reported that IL‐33 is involved in antitumor immunity.^13^ Researchers found that the effects of IL‐33/interleukin 1 receptor‐like 1 (IL1RL1, named as ST2) depend on their expression by certain cells, including ST2^+^CD8^+^ T cells,^14^ helper T cells (Th1 cells), NK cells, and natural killer T cells (NKT cells), and play a role in adaptive as well as innate immune responses.^15^ As an extracellular protein, the IL‐33 binds to the transmembrane‐bound ST2 activates nuclear factor‐κB and mitogen‐activated protein kinases,^16^ and participates in differentiation toward a Th2 phenotype, as well as the activation of mast cells.^17^ Meanwhile, IL‐33 is also vital for the production of Th2 immune mediators. The lymphoid cells that express ST2 included Th2 cells, regulatory T cells, and type 2 innate lymphoid cells (ILC2s).^18^ ST2 has been identified as an IL‐33 receptor, which can induce an IL‐33/ST2 interaction to produce cytokines and activate cells. ST2/IL‐33 signaling has an impact on Th2 cell differentiation or ST2^+^ regulatory T cells during inflammatory bowel disease. IL‐33 is also known to be closely related to tumor survival, growth, and angiogenesis.^19^ Osteopontin (OPN), a highly modified integrin‐binding extracellular matrix glycophospho‐protein, is implicated in tumor progression and metastasis.^20^ Importantly, the stimulation of cell adhesion, migration, tissue remodeling, and tumor metastasis, which can be detected in multiple tumors, is mainly caused by secreted OPN.^21^ OPN appears to be capable of predicting the progression‐free survival (PFS) or outcome of patients with CRC.^22^ The IL‐33 in serum could also be regarded as a predictor of the PFS of gastric cancer patients.^23^ In addition, OPN blockade immunotherapy holds a great potential as a target for colorectal cancer immunotherapy or as an indicator to predict the response of patients with CRC to anti‐PD‐1 immunotherapy.^24^ However, the roles of IL‐33 and OPN in the cetuximab treatment for CRC have not been well defined yet. In our present study, we revealed the changes in IL‐33 and OPN expressions during the course of cetuximab treatment.

Cetuximab is widely used to treat metastatic CRC.^25^ However, there are no indicators of its effectiveness at the early stage of clinical treatment. In the present study, we found that the expression levels of IL‐33 and OPN are different during cetuximab treatment, which is closely related to the effectiveness of cetuximab treatment. The antitumor function of IL‐33 could be initiated by its expression from CD8+ T cells, Th1 cells, NK, and NKT cells. With these findings, we gain a deeper understanding of the mechanisms about cetuximab in CRC treatment.

## 2 MATERIALS AND METHODS

### 2.1 Patients

The scheme of study was reviewed by the Ethics Review Committee of the First Affiliated Hospital, Zhejiang University (Permit number:2018‐969), and the use of human samples complied with the guidelines of the Declaration of Helsinki. The individuals enrolled in the study provided a signed informed consent. Sixty‐three patients and 40 healthy controls subject recruitment were completed at the First Affiliated Hospital of Zhejiang University. Patients and the healthy controls subjects were matched for gender and age. Patients were classified according to the RECIST 1.1 criteria. The effective group (*n* = 50) was defined by radiographic evidence as patients with progression‐free survival (PFS) for at least 6 months. The median PFS for patients of the effective group was 8.4 months (IQR 6.9–14.8). The ineffective group (*n* = 13) was defined as those with PFS less than 6 months. The median PFS for patients of the ineffective group was 3.8 months (IQR 2.4–5.0). The clinical baseline data are shown in Table 1.

**TABLE 1:** The baseline clinical data

Variables	Effective group	Ineffective group
Sex, *n* (M/F)	37/13	5/8
Median age (years, range)	53 ± 8	51 ± 10
White blood cells (mean, 4–10,WBC,10^9^/L)	6.78 ± 0.184	4.78 ± 0.578
Lymphocyte (mean, LC,10^9^/L)	1.28 ± 0.336	2.73 ± 0.063
Metastasis (*n*, %)		
Liver sites only	29 (58%)	8 (61%)
Extra‐hepatic sites	21 (42%)	5 (38%)
Primary tumor (*n*, %)		
Right colon	5 (10%)	4 (30.77%)
Left colon	45 (90%)	9 (69.23%)
Differentiation (*n*, %)		
Highly differentiated	17 (34%)	7 (53.85%)
Low differentiation	33 (66%)	6 (46.15%)

### 2.2 Study design

Cetuximab (Merck) was injected at 250 mg/kg weekly after receiving 400 mg/kg as a loading dose. The monitor of each patient for toxicity weekly and laboratory experiments were undertaken prior to each chemotherapy. Treatment modifications of any drug were decided by the attending physician. Treatment progression was based on RECIST 1.0 criteria, unacceptable toxicities or patient refusal continued until radiological. All patients were assessed by helical computed tomography (CT) (during 28 days prior to starting therapy). A confirmatory assessment was not required. Collection of patients of CRC in 2–4 weeks after the start of cetuximab treatment. Adverse events were recorded.

### 2.3 Cell culture

The cell line (HT‐29) was obtained from cell blank of the Chinese Academy of Sciences (Beijing, China). It was grown in DMEM (Invitrogen) containing penicillin, streptomycin, and 10% bovine calf serum (Biological Industries). The cells were passaged every 2 days at 37°C in a cell incubator of 5% CO_2_.

### 2.4 Nude mouse xenograft assay

The nude mice were obtained from Shanghai SLAC Laboratory Animal Company. All experimental animal procedures were carried out with the approval of Animal Care Ethics Committee of the First Affiliated Hospital, Zhejiang University (Permit number:2020‐1559). For the xenograft assay, Nude mice injection with 1 × 10^6^ HT‐29 cells per mouse into the right flanks of nude mice. Tumor growth was measured by caliper twice weekly and tumor volumes were calculated using the following ellipsoidal formula: Volume = (length/2) × (width)^2^. When volume reached a volume of 200–250 mm^3^, mice were randomly allocated to the control or cetuximab treatment groups and treated for 16 days: Control group (twice weekly, 100 μl of sterile PBS intraperitoneally); cetuximab group (twice a week, 25 mg/kg twice weekly intraperitoneally).

### 2.5 Flow cytometry

The following antibodies were used to stain in the study. The anti‐CD8 (PE), anti‐CD4 (FITC), anti‐CD56 (FITC), anti‐CD137 (PE), anti‐CD107 (APC) (BD Biosciences), and anti‐CD3 (Pacific blue) were purchased from Beckman Coulter. The following antibodies were used: APC Mouse IgG1, k isotype ctrl Antibody (BioLegend), IgG1 (mouse)‐FITC, IgG1 (mouse)‐PE, and IgG1 (mouse)‐PC5 (Beckman Coulter). Data were acquired utilizing FACS Canto II (BD Biosciences) and data were analyzed by using the FlowJo (Treestar).

### 2.6 Peripheral blood mononuclear cells (PBMCs) isolation and culture in vitro

PBMC was isolated after therapy from patient collected at 0 (baseline), and 4 weeks and healthy controls. PBMCs were grown in RPMI medium 1640 containing 10% FBS, and were incubated with cetuximab, IL‐33 (10 ng/ml, Peprotech)^26^ or bovine serum albumin (BSA) of 5 mg/ml up to 48 h.

### 2.7 Cytotoxicity was examined by the real‐time cell analyzer assay (RTCA)

The cell index values were examined using an xCELLigence™ RTCA system (E‐plate 16; ACEA Biosciences). This system is composed by a RTCA impedance analyzer, SP station, 16‐well E‐Plates, and RTCA software. First, 50 µl of media per hole was added to examine the impedance background. Second, 150 μl medium containing 5000 HT‐29 cells were added to each hole. The growth was assessed for 3–4 days by co‐culture with PBMC. After 24 h treatment of cetuximab (5 μM), 250,000 cells of PBMCs in 150 μl medium were added to holes (effector cell/target cell = 50:1).

### 2.8 Immunohistochemistry

Tumor was excised and immediately fixed in a 10% neutral‐buffered formalin at room temperature for 24 h. Then they were dehydrated, paraffin‐embedded, sectioned into 4‐µm thick slices and stained with Ki67.

### 2.9 CCK8 (cell counting kit‐8) assay

For CCK8 assay, cells were added in 96‐wells plates for 24 h treatments of supernatant from the PBMC, which has been treated with IL‐33 and cetuximab. And 10 μl of CCK8 solution was added (Bio‐Rad Laboratories, Inc.) into the cell culture. And then measurements using in a spectrophotometer at 450 nM.

### 2.10 Cell lysis and western blotting

The tumor was lysed with RIPA buffer (Beyotime Institute of Biotechnology) on ice for 30 min and followed by centrifugation at 12,000 *g* for 20 min at 4°C. Next, we perform the quantitation by the BCA method (Thermo Fisher Scientific). The proteins were fractionated by western blot (WB) analysis, and finally detected with chemiluminescent detection reagent (Millipore).

### 2.11 Cytokine analysis

The enzyme‐linked immunosorbent assay (ELISA) (eBioscience) was used for measuring the IL‐4, IL‐6, TNF‐a, and IL‐10 levels. The mature IL‐33 and OPN levels were detected by ELISA Ready‐Set‐Go Kits (R&D Systems) following the manufacturer's instructions. Results were expressed as the number of per ml of plasma or supernatant.

### 2.12 Statistical analyses

Data were analyzed by Prism (GraphPad) software. The difference between two groups was carried out by a student *t*‐test. The comparative analysis date of several group using one‐way ANOVA. The results represent at least three independent experiments and are presented as mean ± SEM. A *p*‐value <0.05 was considered statistically significant.

## 3 RESULTS

### 3.1 Cetuximab treatment induced a higher level of IL‐33 in the effective treatment for CRC patients

**FIGURE 1:**
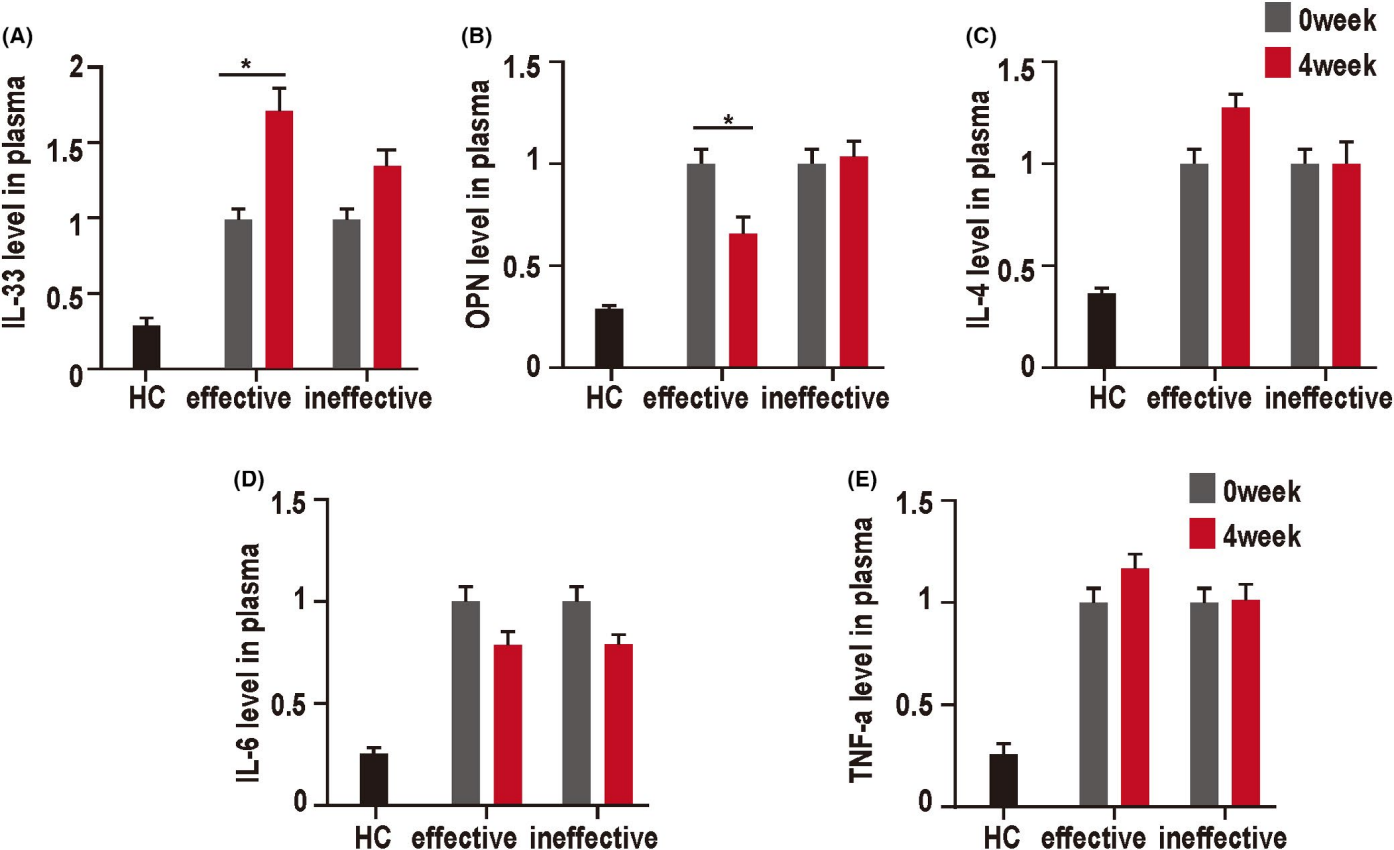
Cetuximab treatment induced the expression of inflammatory factors in the peripheral blood of patients with CRC. Plasma inflammatory cytokine levels were measured using an enzyme immunoassay. (A) The effective group showed a significant increase in IL‐33 at 4 weeks after cetuximab treatment compared with the baseline (0 week). (B) Effective cetuximab treatment decreased the expression of OPN. (C–E) The levels of serum IL‐4, IL‐6, and TNF‐α showed no statistical difference. Healthy Human Plasma (HHP) was used as controls. *p < 0.05. The data represent the mean value ± SEM, as determined using one‐way ANOVA

It has been shown that IL‐33 and OPN are closely related to the development of digestive system tumors; therefore, we wondered whether either of them could represent a promising biomarker as a predictor of prognosis in CRC. TNF‐a, IL‐1, and IL‐4 were classic cytokines secreted by Th1 and Th2 cells, respectively. In addition, it has ever been reported before that colorectal cancer induces an immunological response, shifting the cytokine balance of IL‐4, IL‐6, and TNF‐a.^27^ IL‐1β also plays a critical role in the development of CRC.^28^ Thus, we aimed to determine the effect of cetuximab on those cytokines in the current study. According to the efficacy of cetuximab treatment for 1 year, and based on their progression‐free survival (PFS), we divided the patients with CRC into a cetuximab‐effective group (hereafter referred to as the effective group, PFS >6 months, *n* = 50) and a cetuximab‐ineffective group (hereafter referred to as the ineffective group, PFS ≤6 months, *n* = 13). The levels of the mature IL‐33 and OPN between the effective group and the ineffective group were different after 4 weeks of cetuximab treatment. To address the role of inflammatory factors in the different therapeutic effects, we tested the cytokines in peripheral blood for both the effective group and the ineffective group. IL‐33 levels were significantly higher in the effective group compared with those in the ineffective group after 4 weeks of cetuximab treatment. Meanwhile, effective cetuximab treatment reduced the levels of OPN (Figure 1A,B), while the levels of several other inflammatory cytokines, such as IL‐4, IL‐6, TNF‐α, and IL‐1β did not display significant differences between the effective and ineffective groups (Figure 1C–E; Figure S1). These results suggested that IL‐33 and OPN might be early indicators of the efficacy of cetuximab treatment in CRC.

### 3.2 IL‐33 inhibited the expression of OPN in the effective cetuximab treatment group

**FIGURE 2:**
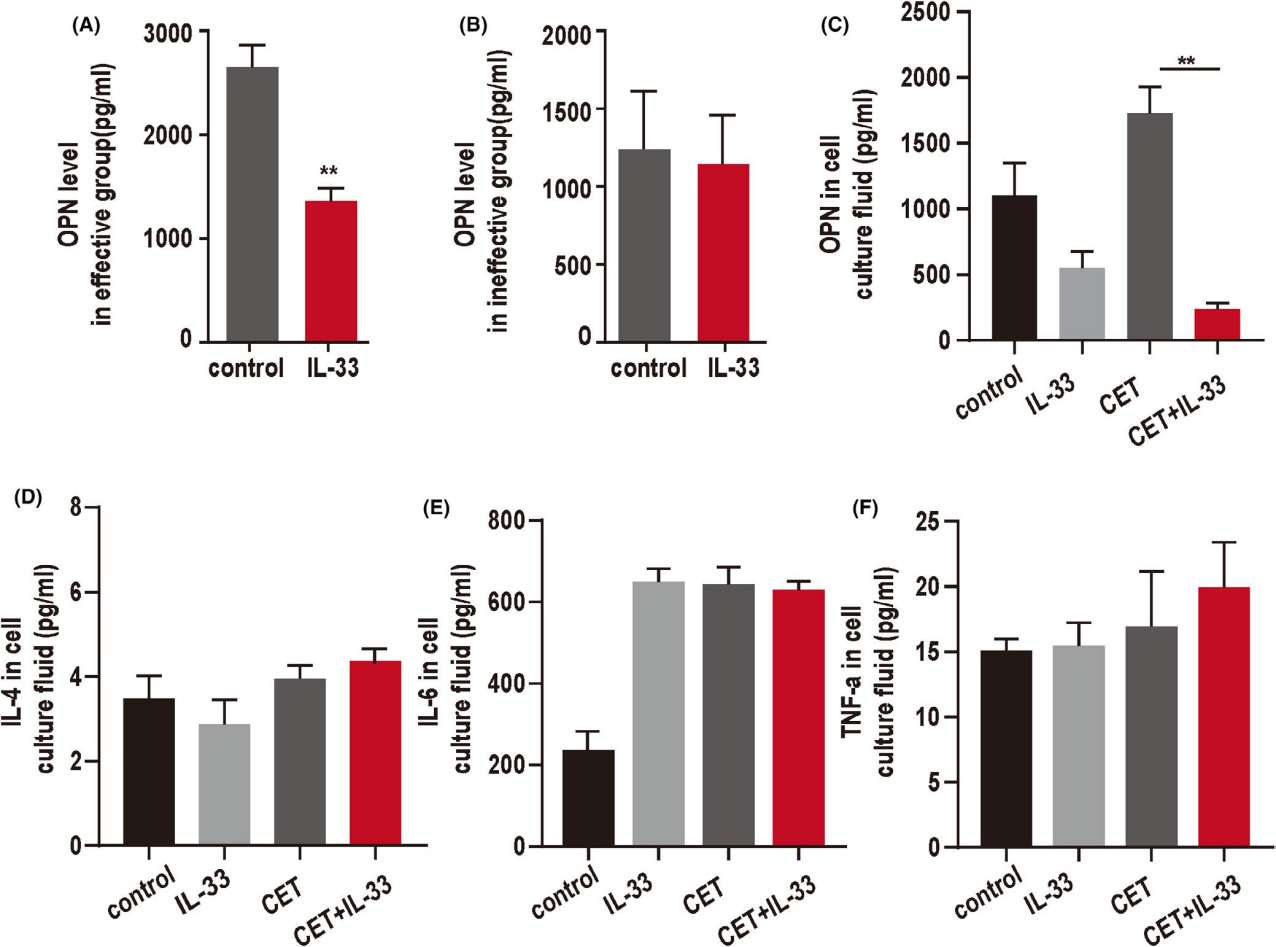
The changes in IL‐33 and OPN levels associated with the early immunotherapeutic effects of cetuximab. PBMCs isolated from patients were stimulated by IL‐33 for 48 h. Cytokine OPN secretion into the culture supernatants was determined using ELISA. For the effective group, the level of OPN was significantly decreased after administration of IL‐33 (10 ng/ml) (A). There was no significant difference in the ineffective group (B). PBMCs from healthy volunteers were stimulated by cetuximab (CET) (5.0 μg/ml) and IL‐33 in vitro for 48 h. Less OPN was produced by PBMCs after CET and IL‐33 stimulation, compared with those stimulated only with cetuximab, but no change was observed for IL‐4, IL‐6, and TNF‐α (C–F). *p < 0.05, **p < 0.01. The data represent the mean value ± SEM, as determined using one‐way ANOVA

Chronic inflammation is the primary risk factor for the prevention of CRC. IL‐33 is a proinflammatory cytokine and plays a key role in regulating colorectal mucosal inflammation. IL‐33 suppressed the expression of OPN in lymphocytes from peripheral blood of patients in the effective group treated with cetuximab for 4 weeks, but made no difference to the level of OPN in the peripheral blood of the patients in the ineffective group (Figure 2A,B). Meanwhile, the level of OPN was decreased by IL‐33 administration in cetuximab‐treated PBMCs from healthy adults, while the effect of IL‐33 alone was not significant (Figure 2C). No significant change was detected for the other cytokines tested (IL‐4, IL‐6, and TNF‐α) (Figure 2D–F). These results indicated that IL‐33 could suppress the production of OPN at the early stage of effective cetuximab treatment.

### 3.3 NK and CD8+T‐cell numbers were significantly increased in the peripheral blood of the effective treatment group, but not in the ineffective group

**FIGURE 3:**
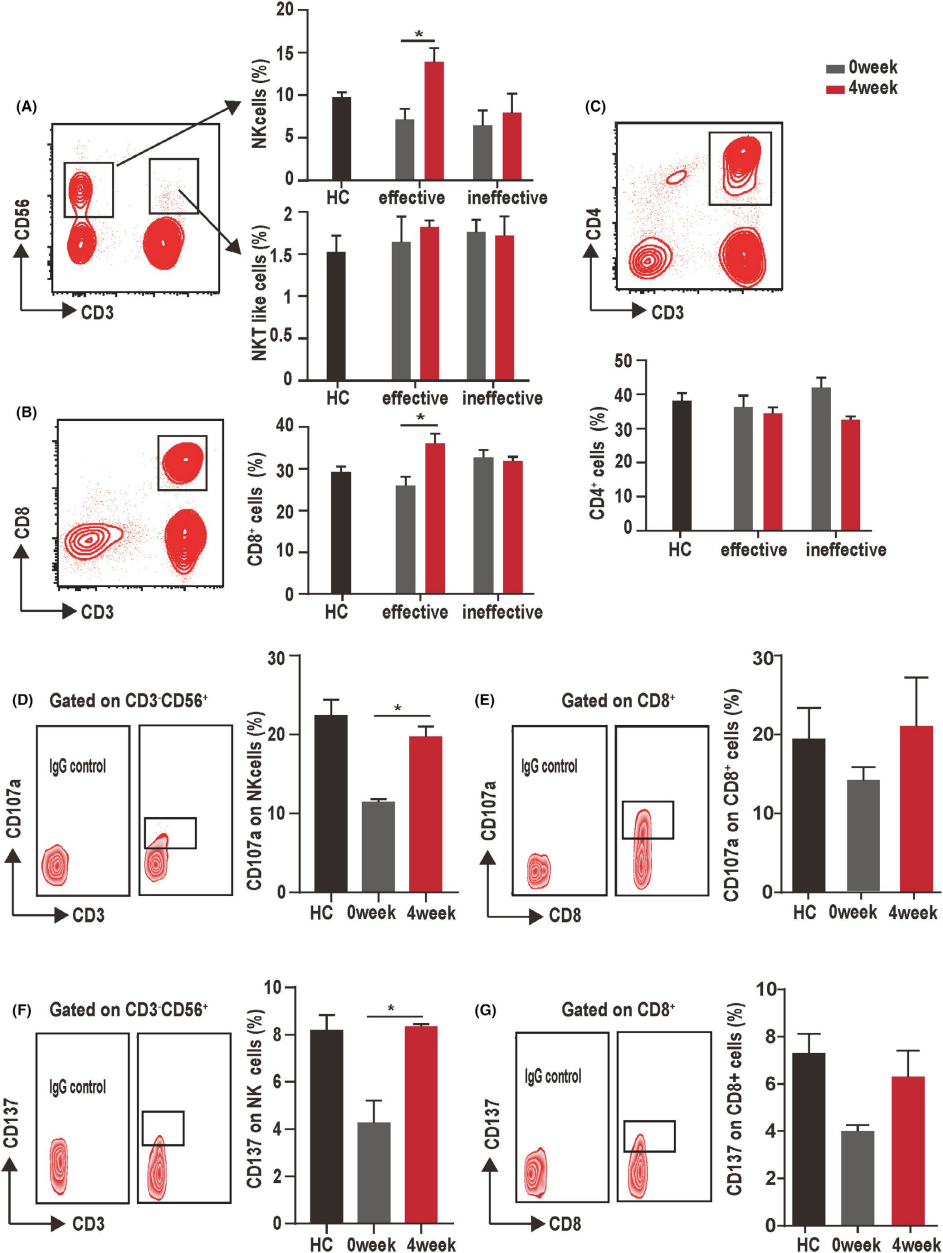
Response of peripheral blood lymphocytes to cetuximab treatment. (A) On the left: Group distribution of NK and NKT‐like cells in the lymphocyte population. On the right: Proportions of NK and NKT‐like cells in the lymphocyte population at 4 weeks after cetuximab treatment. (B, C) On the left: Group distribution in the lymphocyte population (CD3^+^, CD8^+^, and CD4^+^ cells). On the right: Ratio of CD3^+^, CD8^+^, and CD4^+^ cells in the lymphocyte population at 4 weeks after cetuximab treatment. (D, E) On the left: Group distribution of CD107a on NK cells or CD8^+^ cells. On the right: The ratios of CD107a on NK cells or CD8^+^ cells of effective CRC patients at 4 weeks after cetuximab treatment. (F, G) On the left: Group distribution of CD137 on NK cells or CD8^+^ cells. On the right: The ratios of CD137 on NK cells or CD8^+^ cells of effective patients with CRC at 4 weeks after cetuximab treatment. The healthy individuals were used as controls. * *p* < 0.05, **p < 0.01. The data represent the mean value ± SEM, as determined using one‐way ANOVA

NK cells had an increase of the peripheral blood during the early stage of cetuximab treatment in the effective group, but not in the ineffective group. To analyze the contribution of lymphocytes to the effectiveness of cetuximab during CRC treatment, we assessed the peripheral blood lymphocyte subsets of cetuximab‐treated patients with CRC longitudinally using flow cytometry, which was intended to probe NK cells (CD3^−^CD56^+^), NKT cells (CD3^+^ CD56^+^), and T cells. As indicated in Figure 3A,B, a remarkable increase in NK and CD8^+^ T cell frequencies were observed in the peripheral blood during cetuximab treatment in the effective group, while their frequencies did not change significantly in the ineffective group. There was no significant change in the levels of CD4^+^ T cells during the 4‐week treatment period with cetuximab in either the effective or ineffective group (Figure 3C). Collectively, these results suggested that NK and CD8^+^ T cells might play an important role in antitumor immunity in the early stage of cetuximab treatment.

### 3.4 Higher expression of CD107a and CD137 on NK cells occurred in cetuximab treatment effective patients

The studies have identified CD137 and CD107a both are a novel marker of more active cells in CRC,^9^ both of which are implicated in NK cell‐mediated killing of tumor cells. In the present study, we assessed the expression of the CD137 or CD107a in NK cells and CD8^+^T cells from cetuximab‐treated patients with CRC, and elevated expression of CD137 and CD107a were observed in NK cells after effective cetuximab treatment. The levels of CD137 and CD107a in CD8^+^ cells were similar to those in NK cells, but there was no statistical significance in CD8^+^ cells (Figure 3D–G). In addition, no differences were detected between these parameters after ineffective cetuximab treatment (Figure S2).

### 3.5 IL‐33‐induced NK cell activation and ST2 expression after cetuximab treatment in vitro

**FIGURE 4:**
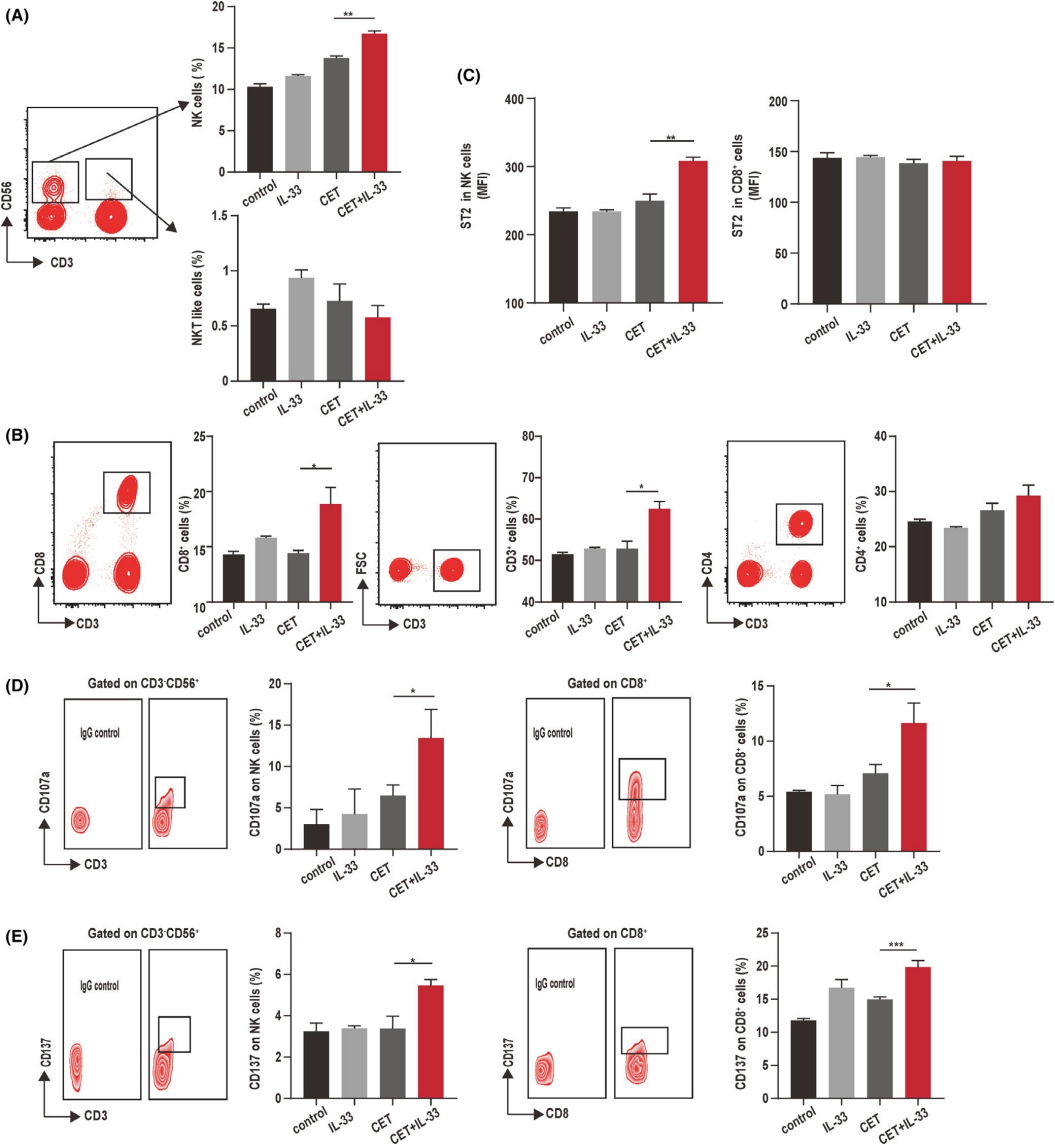
IL‐33‐induced NK cell activation and ST2 expression after cetuximab treatment in vitro. PBMCs from healthy volunteers were stimulated by the cetuximab (CET) (5.0 μg/ml) and IL‐33 (10 ng/ml) in vitro for 48 h. (A) The IL‐33 complexed with CET significantly increased the frequency of NK cells. (B) Ratio of CD3^+^, CD8^+^, and CD4^+^ cells in the lymphocyte population after control (BSA), IL‐33, CET, or CET + IL‐33 treatment. (C) MFI values for ST2 expressed on NK or CD8^+^ cells after control (BSA), IL‐33, CET, or CET + IL‐33 treatment. (D) The ratios of CD107a on NK cells or CD8^+^ cells of healthy volunteers after control (BSA), IL‐33, CET, or CET + IL‐33 treatment. (E) The ratios of CD137 on NK cells or CD8^+^ cells of healthy volunteers after control (BSA), IL‐33, CET, or CET + IL‐33 treatment. *p < 0.05, **p < 0.01. The data represent the means value ± SEM, as determined using one‐way ANOVA

To provide further evidence of the contribution of IL‐33 to the effectiveness of cetuximab treatment, we tested whether IL‐33 could increase the numbers of NK and CD8^+^ cells. A significant increase in NK and CD8^+^T cell frequencies were induced after the addition of IL‐33 to cetuximab‐treated PBMCs, while IL‐33 merely induced a statistically nonsignificant increase of the NK and CD8^+^T cell in the untreated group (Figure 4A,B). Meanwhile, the expression of CD137 and CD107a in CD8^+^ T and NK cells were improved by combined administration of IL‐33 and cetuximab (Figure 4D–E), which was also consistent with the data from cetuximab‐treated patients with CRC. Previous studies revealed that the IL‐33/ST2 axis plays a role in both innate and adaptive type 1 immune responses.^29^ The IL‐33 and cetuximab treatment in our in vitro assays significantly increased the number of ST2^+^ cells among NK cells and the MFI of ST2 staining for ST2^+^ NK cells (Figure 4C). In addition, there were no differences in the concentration of ST2 between effective and ineffective groups (Figure S3). These data suggested that IL‐33 might enhance the activation of immune cells by inducing higher expression levels of CD137 and CD107a after the cetuximab treatment via the binding of IL‐33 and ST2.

### 3.6 IL‐33 exerted a strong antitumor effect through lymphocytes in cetuximab treatment in vitro

**FIGURE 5:**
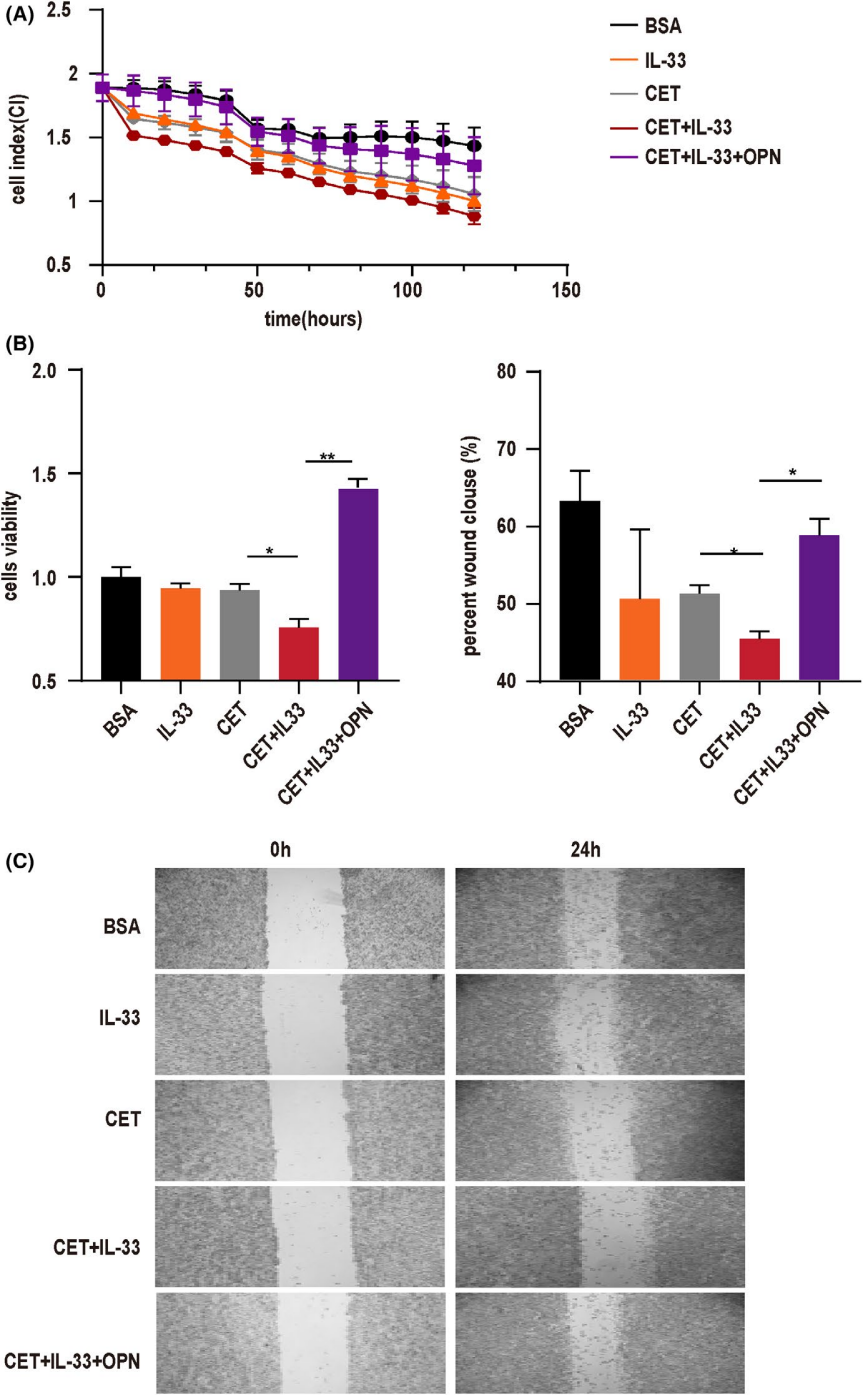
IL‐33 exerted a strong antitumor effect through lymphocytes after cetuximab treatment in vitro. PBMCs from healthy volunteers were stimulated by cetuximab (CET) (5.0 μg/ml), IL‐33 (10 ng/ml) or OPN (5 μg/ml). (A) The cytotoxic activity of PBMCs cells was monitored for using a real‐time cell analysis (RTCA)‐16 system. The cell index represents the cell number and viability. (B) Cytotoxicity of PBMCs was detected after HT‐29 had been co‐cultured with cell supernatants. HT‐29 cell activity was detected using a CCK8 assay by culturing with cell supernatants from different sources. (C, D) Cell migration (scratch) assay. The inhibition of HT‐29 cell growth was examined in cell supernatants from different sources. *p < 0.05, **p < 0.01. The data represent the means value ± SEM, as determined using one‐way ANOVA

To gain further mechanistic insights into the antitumor effect of IL‐33, we examined the antitumor effect of PBMCs under the influence of IL‐33 and cetuximab. Based on the results of cell killing and cell proliferation, we noted that the survival rate of HT‐29 tumor cells following treatment with IL‐33 and cetuximab was significantly lower compared with those treated only with cetuximab. This effect could be counteracted using OPN treatment (Figure 5A,B). Lymphocytes exhibited cytotoxic activity against enterocytes after treatment with IL‐33 and cetuximab in vitro. We found that IL‐33 could promote the antitumor ability of lymphocytes (Figure 5A–C). The separated cell culture supernatant from IL‐33‐stimulated PBMCs could inhibit cell proliferation and migration more significantly compared with stimulation by cetuximab only. The addition of exogenous recombinant OPN could promote cell proliferation and migration to a great extent (Figure 5C,D). It is already known that the regulation of NK cell activity underlies the major mechanism on the antitumor effect of cetuximab.^30^ We performed additional experiments of exogenous recombinant IL‐33 and cetuximab to PBMC (Remove NK cells) or purified NK cell cultures indicate that higher physiological concentrations of IL‐33 may indeed influence the activation of adaptive NK cells in the treatment of cetuximab (Figure S4). Thus, IL‐33 could enhance the antitumor ability of lymphocytes cells together with cetuximab.

### 3.7 IL‐33 and OPN affected the tumor growth process in cetuximab treatment in a mouse tumor model

**FIGURE 6:**
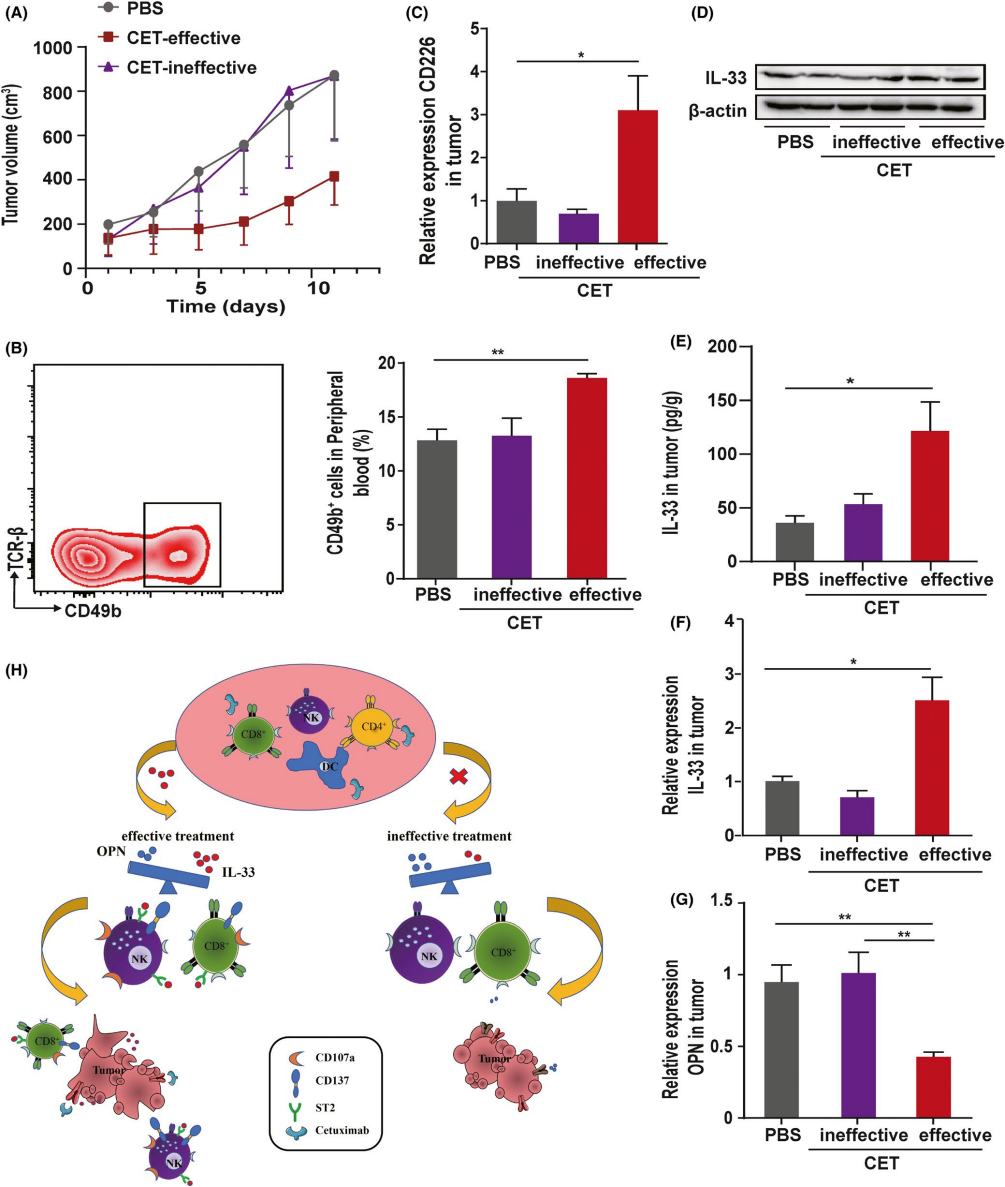
IL‐33 and OPN affected tumor growth after cetuximab treatment in a mouse model. (A) Tumor growth curve in nude mice. (B) Flow cytometry analysis of NK cells from mouse peripheral blood. (C) The expression of CD226 on tumor cells. The level of IL‐33 in tumor cells by western blot (D), ELISA (E), and real‐time qPCR (F). (G) The level of OPN in tumor cells by real‐time qPCR (H). Schematic representation of the major molecular mechanism of IL‐33. *p < 0.05, **p < 0.01. The data represent the mean value ± SEM, as determined using one‐way ANOVA. *n* = 3–6 mice/group

To determine whether the effectiveness of cetuximab against cancer is dependent on IL‐33 and OPN targeting of NK cells, we treated nude mice that had HT‐29 cell xenograft tumors with cetuximab in vivo. Notably, cetuximab monotherapy succeeded in suppressing tumor growth in most of the mice. We then sought to compare the rate of tumor growth among the effective cetuximab treatment group, the ineffective cetuximab treatment groups, and the PBS control group. The rate of tumor growth in the effective cetuximab treatment group (*n* = 4) was markedly lower than that of the PBS control (*n* = 6) and ineffective groups (*n* = 3) (Figure 6A; Figure S5). Cetuximab treatment was capable of altering the expression of the proliferation marker Ki67 in the tumor cells in both the effective and ineffective groups (Figure S6). Comparisons among the three groups showed that cetuximab treatment improved the NK cell ratio in peripheral blood (Figure 6B) and enhanced the infiltration of NK cells in tumors only in the effective group (Figure 6C, Figure S7), while there was no difference observed in the spleen (Figure S8). The levels of CD226, an activation marker of NK cells, were analyzed. We observed a significant increase in CD226 levels in the effective group (Figure 6C). Cetuximab treatment was found to have opposite effects on the level of IL‐33 was increased by cetuximab and that of OPN was decreased (Figure 6D–G; Figure S9).

## 4 DISCUSSION

Along with the changes in the dietary habits in society, the incidence of CRC has been increasing globally.^31^ CRC has the third highest incidence among all cancers worldwide.^32^ However, currently, the efficacy of cetuximab treatment for this type cancer can only be evaluated through imaging and endoscopic examination, and we lack an indicator for the effectiveness of cetuximab at an early stage. Our study revealed a novel link between the levels of IL‐33 and OPN in the peripheral blood and tumor growth, thus suggesting that the two cytokines could be considered as simple and efficient biomarkers to evaluate the effectiveness of early cetuximab treatment of CRC (Figure 6H). Although the significance of IL‐33 has been reported previously for the development of several cancers, the relationship between IL‐33 and effective antitumor medications remained unclear.

Many studies have identified the key role of IL‐33 in tumor growth and the treatment of CRC.^33^ The results in the present study showed that the levels of IL‐33 and OPN in peripheral blood could indicate the efficacy of cetuximab treatment at a very early stage. The administration of recombinant IL‐33 suppressed the expression of OPN from PBMCs in the effective group. The suppressive effect of IL‐33 was also observed in the cell culture assay, where lymphocytes from the peripheral blood of healthy volunteers were stimulated with cetuximab. These results suggested that IL‐33 might exert an antitumor effect by inhibiting OPN expression during early stage cetuximab treatment. Cetuximab is already been approved for the treatment of EGFR‐positive metastatic CRC and also as a treatment for other failed patients with CRC. Therefore, we suggest that IL‐33 and OPN could be developed as a predictive index for the early stage of cetuximab treatment for CRC.

NK cells are important immune cells in antitumor immunity.^34^ Our study showed that the treatment of cetuximab raised the proportion of NK cells and CD8^+^ T cells in the effective group, but there were no significant changes in the ineffective group. A likely explanation for this is that cetuximab treatment induced a significant elevation of NK cells, thus resulting in lysis of tumor cells through antibody‐dependent cell‐mediated cytotoxicity.^35^ Consistent with previous studies, our data extended these findings to evaluate the cetuximab treatment effect. We found that the expression of CD137 and CD107a on NK cells were markedly increased after effective cetuximab therapy. However, there was no significant difference in the expression of CD137 and CD107a on CD8^+^ T cells between cetuximab treatment effective and ineffective patients. Previous studies have found that activated Th1 and CD8^+^ T cells expressed the IL‐33 receptor ST2 transiently, albeit at lower amounts than in Th2 cells.^36^ Recent studies in transplantable solid tumor models using IL‐33 transgenic mice have indicated a direct involvement of exogenous IL‐33 in promoting antitumor CD8^+^ T cell immunity. We deduced that IL‐33 also targets NK cells during cetuximab treatment. Notably, we found that IL‐33 could induce the expression of CD137 and CD107a and the number of CD8^+^ T and NK cells in vitro under stimulation by cetuximab. The expression of ST2 on NK cells was observed in the cetuximab treatment effective patients, indicating that cetuximab possibly induced a significant elevation of IL‐33. High levels of IL‐33 could further bind to ST2 on NK cells, which induced increased levels of CD137 and CD107a in NK cells, as well as increasing the cytotoxicity of NK cells toward tumor cells. The above observation was also consistent with previous findings that IL‐33 enhanced the function of NK cells and CD8^+^ cells in tumors.^37^

The accumulation of OPN can promote the proliferation of tumor cells, which counteracts the antitumor effect of IL‐33 in the tumor microenvironment. In the tumor microenvironment, cytotoxic immune cells were enhanced after treatment with cetuximab. The OPN has been demonstrated to predict low survival rates in CRC, and OPN is capable of inhibiting cell autophagy, possibly by activating the p38 mitogen‐activated protein kinase (MAPK) signaling pathway in CRC.^38^ IL‐33 is an important profibrotic immunomodulatory protein, directly affect eosinophils to an inflammatory state with enhanced production of OPN.^39^ OPN‐deficient mice exhibited a decreased incidence rate and decreased tumor sizes in colon cancer mice model.^40^ OPN is valuable for further study as a potential biomarker for the diagnosis and prognosis prediction in patients with colon cancer in serum or tumor. The results of the present study revealed that the reason for the counteracting effect of OPN against IL‐33 is that the binding between cetuximab‐induced IL‐33 and ST2‐inhibited NK cells from secreting OPN, thus achieving the effect of cetuximab. Given that cetuximab treatment accelerated a cytotoxic response of NK and CD8+ T cells against tumor cells, the results suggested that NK cells could inhibit tumor growth in the nude xenografts after cetuximab treatment. During the treatment of metastatic CRC, the results of present study suggested that IL‐33 could enhance the number of NK cells in the presence of cetuximab. Consequently, the increase in IL‐33 and the decrease in OPN in peripheral blood and tumor tissues suggested IL‐33 and OPN as potential indicators for the efficacy of the early treatment of tumors with cetuximab. Identifying a potential indicator of early stage effectiveness of cetuximab will promote progress in the effective treatment for CRC. PFS was significantly longer for patients of the effective group. Median PFS is 8.4 months for patients of the effective group versus 3.8 months for patients of the ineffective group. The early increase in IL‐33 and the reduction in OPN protein levels also may be correlated with longer PFS. A previous study has also supported that PFS may be a prognostic indicator for cancer patients.^41^ More research is warranted to further determine the mechanism of the combined effect of IL‐33 and cetuximab. The specific mechanism of the interaction between IL‐33 and OPN will be explored in our future study.

## ACKNOWLEDGEMENTS

This work was supported by the National Key Research and Development Program of China (2018YFC2000500), the Key Research & Development Plan of Zhejiang Province (2019C04005), the major National S&T Projects for Infectious Diseases (2018ZX10301401), the Major Scientific Project of Zhejiang Province (2017C03028), the National Natural Science Foundation of China (81472210).

## CONFLICT OF INTEREST

The authors have no conflict of interest to declare about the research.

## Supporting information

Fig S1‐9

## Data Availability

The data that support the findings of this study are available from the corresponding author upon reasonable request.
