# Supplementary material for: Interleukin‐33 as an early predictor of cetuximab treatment efficacy in patients with colorectal cancer
Source: Cancer Med. 2021 Oct 19;10(23):8338–51. doi: 10.1002/cam4.4331 (PMC8633246; doi:10.1002/cam4.4331)
Supplement: Supplementary file 1 — Fig S1‐9 [file CAM4-10-8338-s001.pdf]

S1

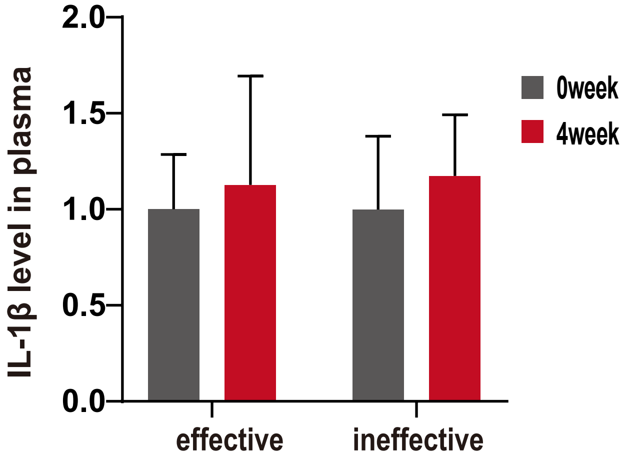

Fig. S1.
The concentration of serum IL-1β shown on statistical difference at 4 weeks after cetuximab treatment compared with the baseline (0week).

S2

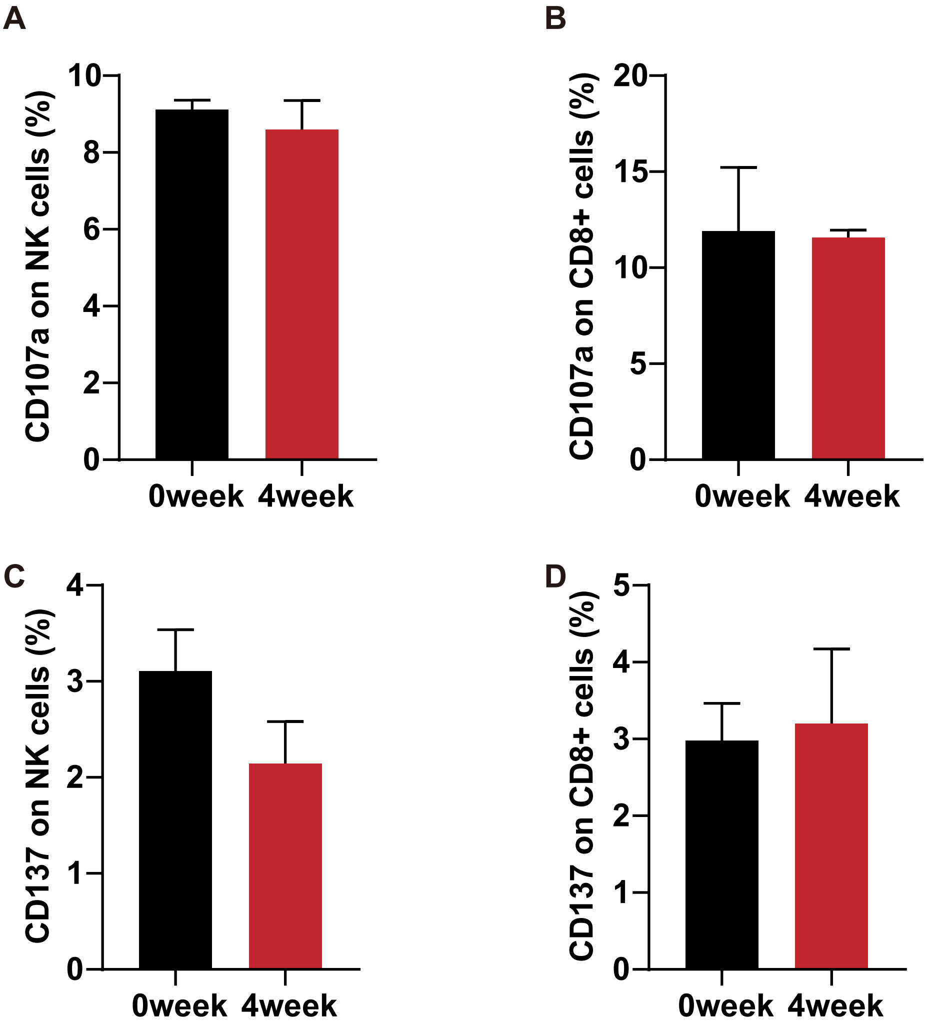

Fig. S2.
A-B:The ratios of CD107a on NK cells or CD8+ cells of ineffective CRC patients at 4 weeks after cetuximab treatment. C-D:The ratios of CD137 on NK cells or CD8+ cells of ineffective patients with CRC at 4 weeks after cetuximab treatment.

S3

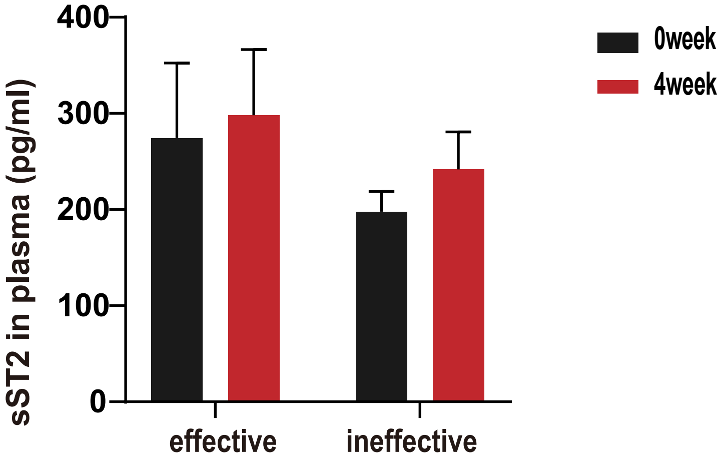

Fig.S3.
The expression of sST2 level in plasma is measured using an enzyme immunoassay of CRC patients at 4 weeks after cetuximab treatment.

S4

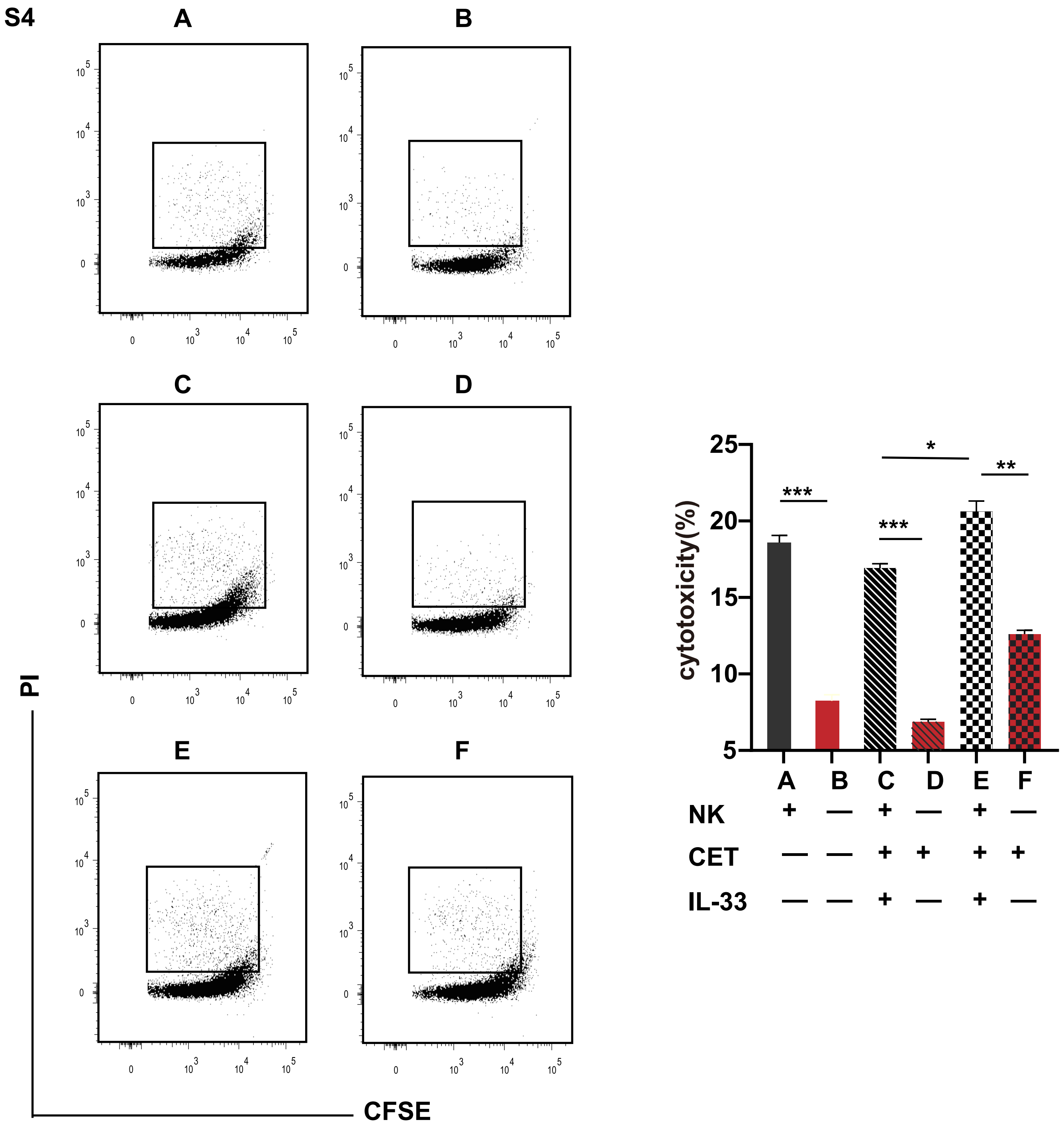

Fig.S4.
NK cells isolated from PBMC and PBMC (Remove NK cells) were stimulated for 24 h in vitro. Cytotoxic assay with target HT-29 cells denoted as the PI-positive rate as analyzed by flow cytometry. * $p < 0.05$, **$P < 0.01$, ***$P < 0.001$.

S5

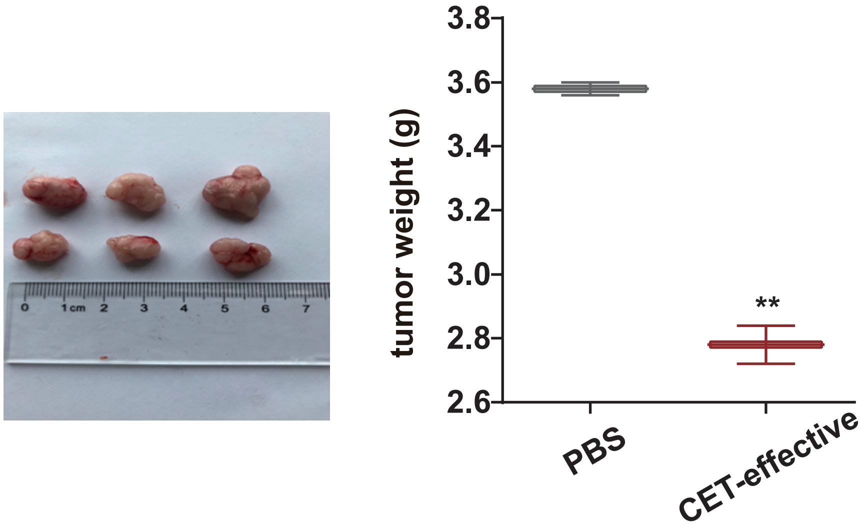

Fig. S5.
Left：Representative images of the nude mice excised tumors; Right：Average weight of tumors in nude mice. **P<0.01.

S6

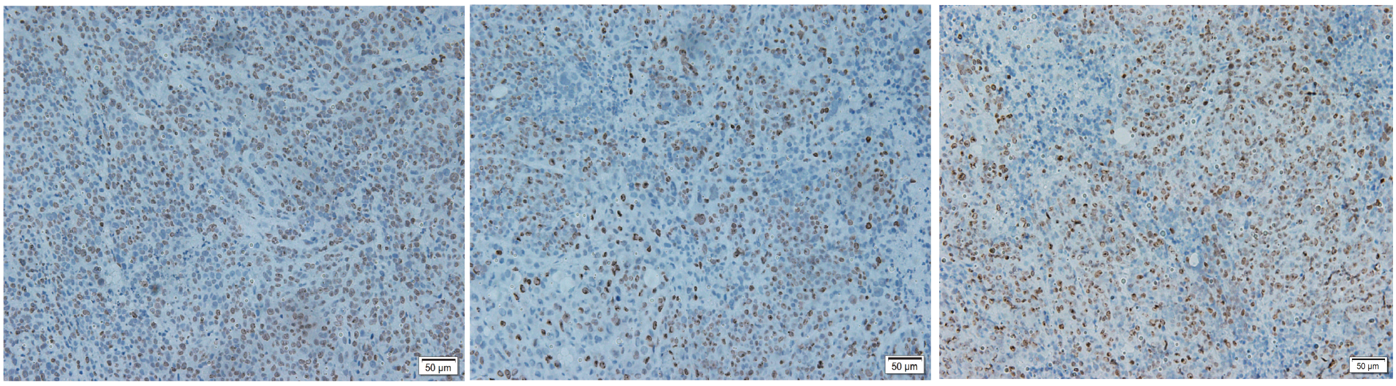

PBS **CET-effectivce** **CET-ineffective**

Fig. S6.
Representative immunohistochemical staining of Ki67 in tumor xenografts excised from nude mice. Original magnification, 200×.

S7

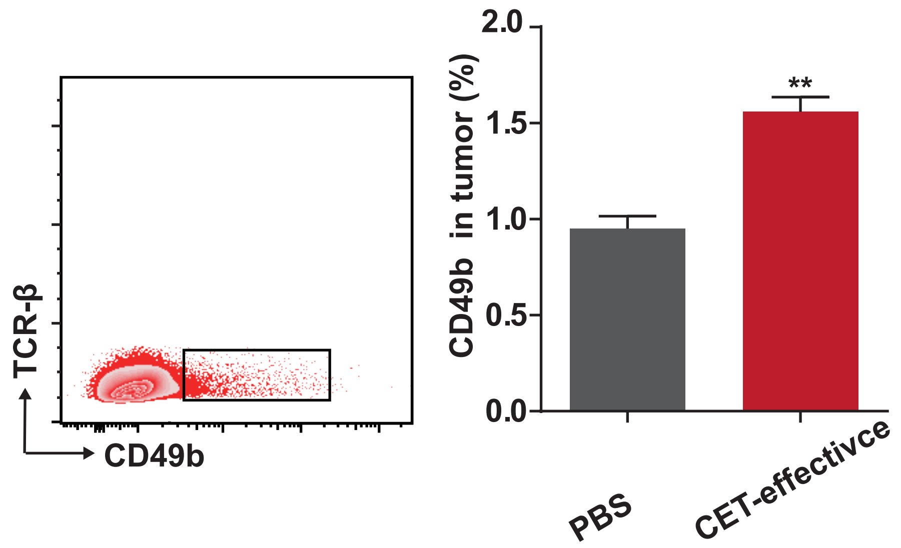

Fig. S7.
Left: Representative dot plots of NK cells in tumor. right: Frequencies of tumor infiltrating NK cells. *P < 0.05, **P<0.01.

S8

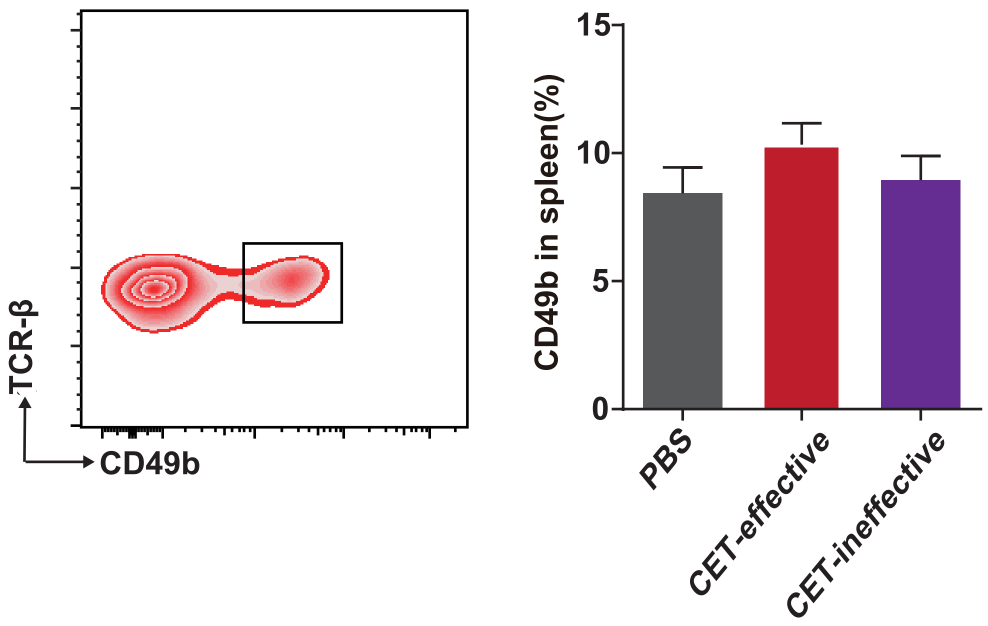

Fig. S8.
Left: Representative dot plots of NK cells in spleen. Right: Frequencies of spleeninfiltrating NK cells.

S9

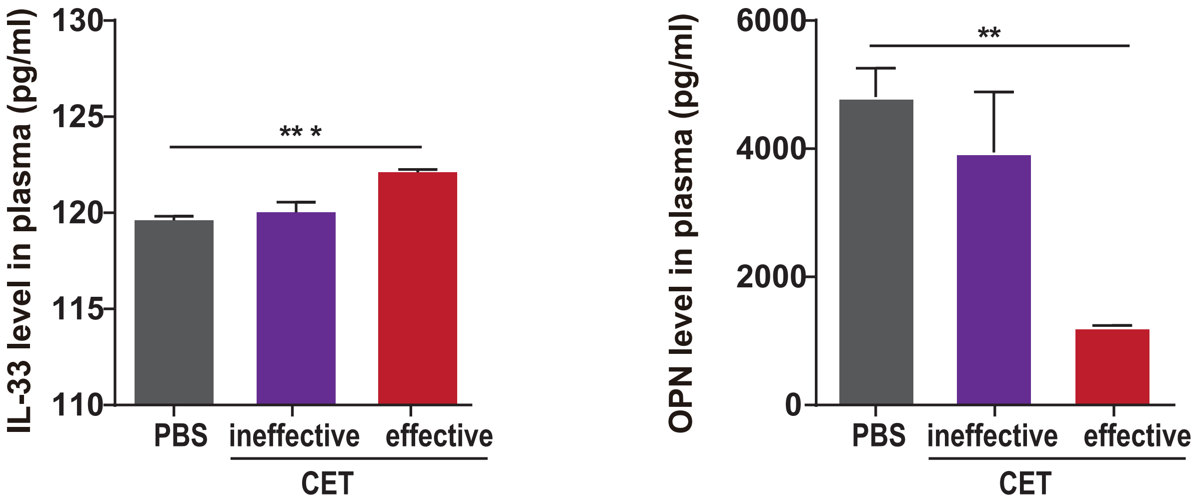

Fig. S9.
Plasma levels of IL-33 and OPN in nude mice. *P < 0.05.
